# Genetically Modified Labeling Policies: Moving Forward or Backward?

**DOI:** 10.3389/fbioe.2018.00181

**Published:** 2018-11-27

**Authors:** Bárbara Juliana Pinheiro Borges, Olivia Márcia Nagy Arantes, Antonio Alberto Ribeiro Fernandes, James R. Broach, Patricia Machado Bueno Fernandes

**Affiliations:** ^1^Biotechnology Group, Federal University of Espírito Santo, Vitória, Brazil; ^2^Retired, Londrina, Brazil; ^3^Penn State Institute for Personalized Medicine, 500 University Dr., Hershey, PA, United States; ^4^Institute for Personalized Medicine, Penn State Milton S. Hershey Medical Center, Hershey, PA, United States

**Keywords:** genetically modified food (GMF), food security, labeling regulation, codex alimentarius commission (CAC), consumers

## Abstract

One of the priorities to address food security is to increase the access of farmers to biotechnology, through the application of scientific advances, such as genetically modified organisms and food (GMF). However, the spread of (mis)information about their safety strengthens the clamor for mandatory GMF labeling. This paper provides an overview of food labeling policies, considering the principles suggested by the Codex Alimentarius Commission, and analyzes the consequences for the world food security of the Brazilian labeling policies compared to developed countries. We discuss the discriminatory application of GMF mandatory labeling in the absence of any scientific evidence as it has the potential of causing social harm and jeopardizes research, production, and distribution of food and consumers' right to information.

## Introduction

Food security depends on political will and government action and should be based on scientific evidence. This security is not a final or static achievement. On the contrary, it presents constant challenges, which requires frequent adjustments to be met.

The Food and Agriculture Organization of the United Nations, FAO, defines food security as the condition “when all people, at all times, have physical, social, and economic access to sufficient, safe and nutritious food which meets their dietary needs and food preferences for an active and healthy life” (FAO, 2017). Although the number of undernourished people has been dropping since 1990, 795 million people still suffer from food insecurity (FAO, 2015). The concept of food security comprises food safety which not only incorporates food quality but also includes health aspects to eliminate food that causes sickness or intoxication. Food safety represents an important component of food security and should receive even more attention since 3 million people per year likely died from food and waterborne disease prior to 2010 (FAO, 2010b).

In this context, public decision-makers have an important role in promoting food security. An example of a relevant international initiative is the Global Forum on Agricultural Research (GFAR), a worldwide network created with the responsibility to define directions for agricultural research. This forum mobilizes partners in the academy, government, and society to strengthening research and extension systems all over the world, especially in developing countries (GFAR, 2017). Not only international but also local, policymakers and public decision-makers should provide a clear and honest dialogue with society. This position can contribute by supporting a decision-making process, at individual and collective levels, that end up affecting the destiny of the global food security. Such decisions must be built and based on a scientific framework, which will ultimately inform regulation (Małyska et al., 2014). To achieve that goal, lay people must have a secure means of obtaining reliable information. However, genetically modified organisms (GMO) and derivatives (for example, food) usually evoke controversial and radical opinions. Few debates are focused on providing transparent and substantial information (McHughen and Wager, 2010).

Investments in biotechnology products have the potential to enhance the food supply, especially related to nutrition, taste, price, and reduction of food waste. Actually, it has determined that the non-use of GMO technology in agriculture would have negative consequences for the welfare of the US and global economy due to reduced yield and increased environmental damage and food prices (Taheripour et al., 2016). Although, the current access availability to media, especially the web, does not ensure access to reliable information that would support a sensible and autonomous decision. Instead, such easy access facilitates the spread of (mis)information about the safety of genetically modified food (GMF) and derivatives (Capalbo et al., 2015). This has led to mandatory GMF labeling justified by consumer opposition (Kaneko and Chern, 2005).

Nowadays, discussions about GMF labeling in the US raised an opportunity to investigate GMO regulatory system (Kling, 2014) and its costs (Mclean et al., 2012), which needs revisions aligned with the scientific advances (Strauss and Sax, 2016) not only in the US but also in countries responsible for the world food supply. This is particularly relevant to Brazil given its role in the world's food production and exportation (FAO, 2015; OECD FAO, 2015). As a matter of fact, the Brazilian National Congress (Brazilian Parliament is composed of the Chamber of Deputies and the Federal Senate) started addressing this topic in 2008 but has not reached a reasonable consensus, which is under discussion through Bill n° 34/2015 and Bill n° 4908/2016.

Here, we review GMF labeling in the Codex Alimentarius Commission (CAC) guidelines and in the Cartagena Protocol on Biosafety (CPB). To set an example, we focused on food labeling policies in Brazil compared to developed countries, which have similar capacity of GMF production, for their relevance in the global food supply. We also analyze consumer's right to information in the context of food labeling policies and its potential impact on global food security. Finally, we present suggestions to update the Brazilian regulatory framework in accordance with the international harmonization.

## Principles and roles of food labeling

The international standardization Codex Alimentarius Commission (CAC) has as its main objectives: protecting the health of consumers and ensuring that regional and international food trade employs fair practices (GFAR, 2017). The CAC establishes principles and guidelines for food safety assessment of GMF in the document “Food derived from modern biotechnology.” The first part addresses risk analysis, which covers risk assessment, risk management, risk communication, consistency, capacity building, information exchange, and review processes. Risk assessment includes safety assessment based on an appreciation of science-based multidisciplinary data: “Risk assessment should take into account all available scientific data and information derived from different testing procedures, provided that the procedures are scientifically sound and the parameters being measured are comparable” (WHO, 2009).

The CAC addresses labeling issues only in the context of risk management: if risk assessment identifies no significant risk, labeling is not needed. Therefore, labeling should be considered only if both the risk is present and the GMO is approved (CAC, 2011b). It suggests “food labeling conditions for marketing approvals and post-market monitoring” in order to track products, for example, if it is strictly related to “potential consumer health effects” (WHO, 2009).

Indeed, a document entitled Compilation of Codex Texts Relevant to Labeling of Foods Derived from Modern Biotechnology corroborates this argument: “[this document] is not intended to suggest or imply that foods derived from modern biotechnology are necessarily different from other foods simply due to their method of production” (CAC, 2011a). Moreover, CAC recommends applying the same rules concerning food labeling regarding the allergenic potential to both biotechnology-derived and products not obtained by modern biotechnology (WHO, 2007). Finally, the “Review Processes” suggests that analysis and risk management should be “evaluated and reviewed as appropriate in the light of newly generated scientific data” (CAC, 2007; WHO, 2009).

The Cartagena Protocol on Biosafety (CPB), part of the Convention on Biological Diversity, expresses a major concern with public awareness of living modified organisms (LMOs) issues. This Convention formulated a survey to collect data about “public awareness of issues concerning the safe transfer, handling, and use of living modified organisms in the context of the Cartagena Protocol on Biosafety” that parties, i.e., governments adopting the protocol, should promote. These parties should also participate in regional networks on public awareness, education, and participation concerning LMOs (Biosafety Clearing-House., 2012). Nevertheless, this protocol does not address the marketing of GMF. Rather, the protocol's objective is safe transferring of LMOs specifically focusing on transboundary movements (Secretariat of the Convention on Biological Diversity, 2005).

## Food labeling policies in brazil

The Constitution of the Federative Republic of Brazil (CRFB) is the supreme law in Brazil but contains no specific topic on food labeling. CRFB covers general principles governing the economy and health surveillance, and since foods are products placed on the market, these products should be subject to these principles.

Regarding the general principles governing economy, a pivotal issue is the consumer's right according to Federal Law n° 8.078/1990. This Law, also known as Consumer Protection Code (CDC), establishes the right to information as a primary right of the consumer. This right is characterized by the adequacy and clarity of the information offered about different products and services. It also implies that the label (of products and services) must correctly specify the quantity, characteristics, composition, quality, taxes, price, and risks.

The regulations related to health surveillance are more detailed and establish the rules for food labeling. The Brazilian Health Surveillance Agency (ANVISA), the Ministry of Agriculture, Livestock and Food Supply (MAPA), and the National Institute of Metrology, Quality, and Technology (Inmetro) are the main government institutions responsible for enacting the rules on food quality and control. In addition to the aforementioned institutions, the National Technical Commission on Biosafety (CTNBio) is a multidisciplinary collegiate body responsible for establishing technical safety standards and advice on GMO and derivatives, including GMF. CTNBio is a subsidiary body of the Ministry of Science, Technology, Innovation and Communication, and is composed of 27 members and their substitutes. Its diverse composition demonstrates the democratic nature of this Board. The members are not only experts of widely recognized scientific knowledge, vouched for by scientific societies and the academic community, but also representatives from the civil society in the consumer protection, health, environment, biotechnology, agriculture, family farming, and health professionals (Souza et al., 2013).

Rules on food labeling can be divided into two groups, namely, general rules of labeling and rules of nutritional information. The general rules of labeling gather obligatory requirements for all foodstuffs. The rules of the nutritional information aggregate the instructions for displaying analytical nutritional composition and contents on the label. The main Brazilian rules are delineated in legal documents as shown in Table 1.

**Table 1:** Brazilian legal documents about general food labeling and nutritional information.

**General rules of labeling**	**Rules of nutritional information**
Decree-Law n° 986/1969 ANVISA RDC n° 259/2002 (modified by ANVISA RDC n° 123/2004)	ANVISA RDC n° 359/2003 ANVISA RDC n° 360/ 2003 (modified by ANVISA RDC n° 163/2006 and rectified in 2013)
Inmetro Ordinance n° 157/2002 Law n° 10.674/2003 ANVISA RDC n° 123/2004	ANVISA RDC n° 163/2006 ANVISA RDC n° 31/2012 ANVISA RDC n° 54/2012 ANVISA RDC n° 26/2015

The first group includes ANVISA Resolution of the Collegiate Directorate (RDC) n° 259/2002. This Resolution approves the Technical Regulation on Food Labeling Packaged (modified by ANVISA RDC n° 123/2004). In addition, Decree-Law n° 986/1969 establishes basic rules about food. The Resolution and Decree-Law together represent the core of the general rules of food labeling. They detail the requirements for food labeling, namely, description of the product (name/brand, quality, nature, and food type), list of ingredients, liquid contents, identification of the source (data of the manufacturer, producer, fractionators or holder/owner of the brand), food registration number with the government competent agency, lot identification, and expiration date. Additionally, the importer must be identified in the case of imported products and the instructions for preparation must be given in the case of products not ready to eat or drink.

Inmetro Ordinance n° 157/2002 approves the Metrological Technical Regulation that details the requirements for describing the content of the products. For example, this regulation specifies the volume and mass measurement units that must be used.

ANVISA RDC n° 360/2003 is the primary Resolution addressing the second group of food labeling rules. It approved the Technical Regulation on Nutritional Labeling of Packaged Food, which became mandatory for nutrition labeling. The other Resolutions establish the Reference Values Table for Food and Beverage Packaged for Nutritional Labeling (ANVISA RDC n° 359/2003), the Nutritional Labeling of Non-Alcoholic Beverages Sold in Returnable Packaging (ANVISA RDC n° 31/2012) and the Technical Regulation on Common Market of the South (MERCOSUR) Additional Nutritional Information (ANVISA RDC n° 54/2012), a facultative nutritional information intended to satisfy requirements of the MERCOSUR.

All types of foodstuffs, including GMF and derivatives, must follow the rules for nutritional information established in the second group of regulations. In addition, there is a specific Law (n° 10.674/2003) enforcing that marketed food products identify on the label the presence of gluten for control of celiac disease. It requires the use of the terms “contains gluten” or “gluten-free” in the labels of industrialized foods, whether or not they are GMF. Another ANVISA Resolution (ANVISA RDC n° 26/2015) lists the main ingredients known to cause allergies and establishes requirements for mandatory labeling of the foodstuffs that contain (or may contain) these ingredients, independently of the methods or techniques used to produce the food and its ingredients. The purpose of this regulation is to protect the consumers and prevent health damage caused by potential allergenic ingredients present in GMF or non-GMF.

Neither of the two groups of Regulations requires specific mandatory labeling for GMF and derivatives. The Regulations only specify what information food labeling must obligatorily exhibit and how that information must be presented (Table 2).

**Table 2:** Obligatory information required by Brazilian food labeling regulations and consumer's right legislation.

**Obligatory information for industrialized/packed and** ***in natura*** **food (when in packages)**	
Description of the product list of ingredients liquid contents (measure standards) identification of the source food registration number lot identification expiration date importer's identification, if applicable	Instructions for preparation, if applicable nutritional information ingredients that cause allergies presence or absence of gluten (industrialized food) taxes price risks

Decree n° 4.680/2003 was the first GMF labeling Regulation issued in Brazil. It is not part of the health surveillance regulations, but addresses, instead, the right to information about food and food ingredients intended for human or animal consumption containing or produced from GMO (or GMF). These types of foodstuff are considered by the decree to share common characteristics by the fact that they are produced through modern biotechnology techniques. Despite the fact that the CAC does not treat GMF as different from other foods simply due to the method of production (CAC, 2011a), these products are subject to the specific regulation (Table 3).

**Table 3:** Regulation on GMF labeling currently in force in Brazil.

**Legislation**	**Requirements**
Decree n° 4.680/2003	Both industrialized and *in natura* foodstuff which contain more than one percent of GMOs should be labeled. The label must include the name of the gene donor species. If a food is animal material and the animal was fed GMO feed, it must also be labeled.
Ordinance n° 2.658/2003	Prescribes the use of the letter “T” in the center of a yellow triangle with black outline as a GM symbol in the label.
Interministerial Normative Instruction n° 1/2004	Establishes the Technical Regulation on Food Labeling and Food Ingredients that contain or are produced from GMOs.
Law n° 11.105/2005 (also known as Biosafety Law)	States that information about GM nature must be included on the label of foods and food ingredients intended for human or animal consumption, but does not specify how this should be done.
Decree n° 5.591/2005	Regulates the Biosafety Law, does not specify GMO labeling procedures and merely reproduces the text of the law (Article 91).
Decree n° 6.041/2007	Establishes a Biotechnology Development Policy and creates the National Biotechnology Committee. One of the guidelines of the policy is the creation of regulation of conformity assessment, including labeling.

This body of Regulations seems to have exceeded its mandate since neither the previous Biosafety Law nor the regulatory decree (Decree n° 1.752/1995) requires specific GMF labeling procedures. Therefore, Decree n° 4.680/2003 established the first mandatory GMF labeling without a supporting law. In a democratic republic government, regulations must not be issued in the absence of a law and the decree should be nullified in court. However, no cancelation process occurred and a new Biosafety Law (Law n° 11.105/2005) was issued and included the requirement as mandatory.

While the current Biosafety Law (Law n° 11.105/2005) and its Decree include GMF labeling requirements, perhaps as a result of concern about GMO safety by some groups in Brazil at the time, the CPB and other international agreements contain no rules on labeling of GMOs or GMF. The only exception corresponds to the transport of living modified organisms (LMOs) across countries. The Protocol recommends labeling the loads that contain LMOs as part of the risk assessment. Risk assessment is a tool for safe transfer, handling, and use of LMOs, specifically focusing on transboundary movements (Secretariat of the Convention on Biological Diversity, 2005). Hence, this sort of labeling is different from the one proposed for GMF in Brazil; this is a clear example of current misunderstanding and misuse of the Precautionary Principle stated in the Protocol (Tagliabue, 2016). As mentioned, CAC states that food labeling may be included in the risk management measures, it is not obligatory (CAC, 2008).

In Brazil, CTNBio is the body responsible for carrying out the risk assessment. This commission technically analyzes every GMO or derivative, not only LMOs. If a risk is deemed to exist, the commission establishes criteria for monitoring the GM product and derivatives. The items requested by CTNBio to the GMO developer are described in its normative instructions and resolutions, following the suggestions of the CPB and the CAC. Only after the commercial release approval of the product would a post-market monitoring proposal and the monitoring reports be necessary. CTNBio has received no report of adverse effect to date. However, if adverse effects were observed, the analysis should be done on a case-by-case basis. CTNBio's technical advice supports decisions on the market release of GMOs. The National Biosafety Council is responsible for the final decision, taking into account the socioeconomic convenience and the national interest.

In addition to the Biosafety regulatory framework, ANVISA ensures measures for protecting consumers by establishing mandatory labeling to food containing allergens whether or not these products are biotechnology-based. ANVISA's rule harmonizes to the concepts embodied in the CAC's documents (WHO, 2007; CAC, 2011a).

**Figure 1:**
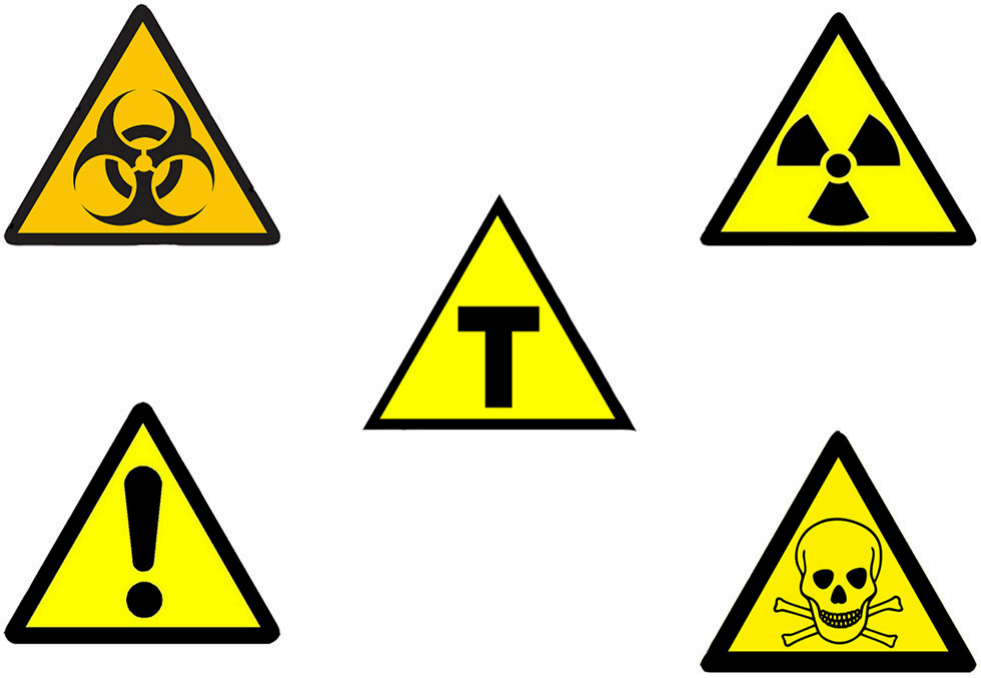
Brazilian GMF and international hazard symbols. Brazilian GMF symbol (the letter “T” in the center of a yellow triangle with black outline—center), biohazard symbol (top left side), radiation hazard symbol (top right side), toxic hazard symbol (bottom right side), and warning hazard symbol (bottom left side), displaying the similarity between them that can promote miscommunication in food labeling. Source: Authors adapted from Andre, 2010; Kartoffel07., 2013; Grupo Transgênicos, 2016 authorized under the Creative Commons Attribution-Share Alike 4.0 International, Commons Attribution-Share Alike 3.0 and the Commons Attribution 4.0 International, respectively.

Lastly, the Brazilian obligatory symbol to identify GMF employs a shape (triangle) and colors (yellow and black; Figure 1) that, according to the United Nations Economic Commission for Europe (UNECE) and International Standard Organization (ISO) are associated with the idea of alert, attention, and danger (UN, 2011; ISO, 2013). This misuse of visual communication in food labeling promotes public mistrust and a priori negative judgment of the nutritional value of the food. The use of some colors, which are usually associated with alerts, implies that consumers must be warned about generic safety hazards associated with the process of genetic modification. However, there are no generic safety hazards, meaning that those colors should not be employed.

**Figure 2:**
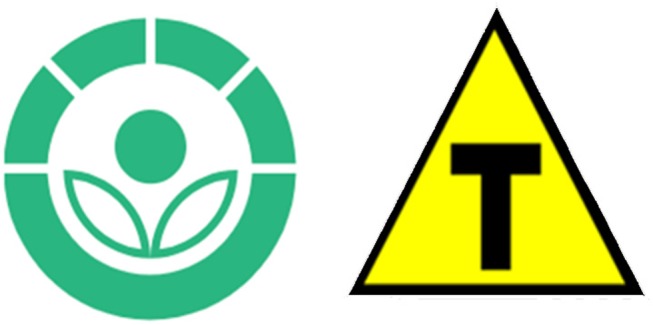
Radura and the Brazilian GMO symbol. Radura (left side) is the international symbol for food that has been treated with ionizing radiation. The obligatory symbol used to identify GMF in Brazil (right side) is related to international visual communication for hazardous products. Source: Authors adapted from United States Department of Agriculture (2006), in the public domain; and from Grupo Transgênicos, 2016 authorized under the Creative Commons Attribution-Share Alike 4.0 International.

ANVISA RDC n° 21/2001, for example, establishes a mandatory label for foodstuffs exposed to ionizing radiation. This process should not be used as a substitute for good practices of food manufacturing. Its use is justified only if specific technological requirements are fulfilled and for beneficial application and protection of consumer health (CAC, 2013). The label must indicate that the food product has been irradiated and the international symbol, called Radura, should be placed in the package (United States Department of Agriculture, 2012). Radura is a non-alarming picture that employs light colors (green and white) and shapes (similar to leaves and flower; Figure 2) instead of inducing hazard warning as the use of a yellow triangle for GMF/GMO in Brazil.

Currently, there are two bills under discussion in the Brazilian National Congress. The first, Bill n° 34/2015, recommends mandatory labeling for foods containing or produced from GMO with more than 1% in the final composition, and the withdrawal of the GM symbol but it determines the use of the terms “contains trangenic.” The second, Bill n° 4.908/2016, proposes the use of pictures that represents the risks of GMF, such as the ones placed in the cigarette pack by WHO guidelines.

## Food labeling policies concerning GMF in selected countries

Australia, United States of America (US) and Canada join Brazil in leading positions in the exportation of food (WTO, 2016), being relevant players in the world's food supply. Therefore, we have selected these countries to inquire about their food labeling policies focused on GMF.

### Australia

In Australia, GMOs are regulated through the Gene Technology Act 2000 (also known as Commonwealth Act) and the Gene Technology Regulations 2001. Commonwealth Act includes measures relating to general labeling in the numerous conditions that may be prescribed or imposed in order to get the license in relation to GM products that are derived from a GMO (Australia, 2016b,c).

The Commonwealth Act contains extensive monitoring, compliance and enforcement powers at a national level but considering regional interests, Each State and Territory, including Western Australia (WA) had legislation equivalent to the Commonwealth Act. The situation of WA was particularly interesting because this state had forbidden growing GM crops under the Crops Free Areas Act 2003 (GMCFAA). This legislation was not related to safety and health assessments; on the contrary, it relied on marketing grounds. But in October 2016, the WA Government removed this potential barrier through repealing the GMCFAA. Currently, agricultural producers in the state can access GM crops approved for commercial release in Australia (Government of Western Australia, 2015).

Concerning food, the country has enacted the Food Standards Australia New Zealand Act (FSANZ Act) in 1991 but it does not detail GMF policies. This act only indicates matters that may be included in standards and their variations, including any information about food labeling, promotion, and advertising. FSANZ Act established an independent statutory agency, FSANZ, responsible for developing standards that regulate the use of ingredients, processing aids, colorings, additives, vitamins, and minerals in food, including food labels (Australia, 2016a).

The Australia New Zealand Food Standards Code is the instrument that contains the standards. The Code is systematically organized in six parties. Standard 1.2.1 belongs to part 2. It demands mandatory labeling establishing the general rule: “information relating to foods produced using gene technology” is required on foods that have novel DNA or novel protein. This novelty means that the DNA or the proteinchemical sequence or structure in GMF differs from those present in the counterpart food. Standard 1.5.2 belongs to part 5 (“Food requiring pre-market clearance”) and details GMF labeling. This standard describes which the exceptions of the general rule are. Hence, some types of GMF escape labeling, including food to be eaten immediately and highly refined food, such as oils and sugars. Besides, GM food additives and processing aids must be labeled since GM DNA or protein are present, and GM flavors is only required to be labeled if they represent more than 0.1% of the food. Finally, in the case of occurring accidental “contamination” of the food by a GM ingredient or processing aid, labeling is required if the contamination is more than 1% of the food (FSANZ, 2018).

### United states of america (USA)

The first US regulation of activities involving GMOs was published by the White House Office of Science and Technology Policy (OSTP) in 1986. The Coordinated Framework for the Regulation of Biotechnology (CF) was last updated in 2017 and explains the regulatory jurisdictions of the major regulatory agencies. These agencies of the US government are responsible for the matter: the United States Department of Agriculture/Animal and Plant Health Inspection Service (USDA/APHIS), the US Environmental Protection Agency (USEPA) and the Food and Drug Administration (FDA). According to the nature and characteristics of the product and its application, it will be under a specific regulatory path and relevant procedures. The FDA is responsible for protecting the public health by ensuring the safety, efficacy, and security of food, including GMF (OSTP, 2017).

Until 2016, foods containing GM ingredients available on the US market did not need to be labeled since the FDA had determined that these foods were “substantially equivalent” to their non-GM counterparts. Therefore, Americans have been consuming products, including GMF such as corn, oils, sugars, but they had not the information about the presence of GMOs in these products (Fernandez-Cornejo and Caswell, 2006).

At that time, the FDA has recommended voluntary labeling indicating whether foods are or are not of genetically modified origin, so long as such labeling was truthful and not misleading. Therefore, the FDA published a guidance to “food manufacturers to ensure that labeling terminology concerning the use of modern biotechnology (…) be accurate and consistent and that the integrity and meaning of scientific terminology be preserved to help ensure clear communication in food labeling.” So, the use of the terms “GMO-free,” “GE free,” “does not contain GMOs,” “non-GMO,” was discouraged (FDA, 2015).

The US has a history of adopting fewer restrictions on the regulation of products derived from modern biotechnology. However, in 2016, a law that mandated the development of a National Bioengineered Food Disclosure Standard was passed. This law establishes GMF mandatory labeling and harmonizes legislations in the country. It was a reaction to the passage of a GMF labeling law by the State of Vermont, with the prospect that any or all of the remaining 49 states might enact its own state law. Considering the potential troubled outcome of 50 incompatible labeling regimes in the country, authorities decided to pass a federal law and unify an American marketing standard. So, the passage of this law refers to marketing topics and not to safety claims.

USDA's Agricultural Marketing Service (AMS) was assigned to establish a national standard containing requirements for labeling of human food products derived from biotechnology (US Government, 2016). AMS has 2 years to settle the standard and the procedures necessary for implementation. USDA has proposed rule questions and received contributions from the interested stakeholders. Although a draft standard has been released, the rulemaking process is still ongoing and will finally provide a publication of the final text summarizing and responding to the public, including revisions and providing an effective date (AMS, 2017).

### Canada

Canadian regulatory framework encompasses the Foods and Drugs Act, Food and Drug Regulations, Consumer Packaging and Labeling Regulations, and their amendments. Foods and Drugs Act establishes general rules for all types of foodstuff. Food and Drug Regulations establishes criteria and detailed rules for the development of any activity concerning food and drug.

In the Regulations, there is a specific section named “Division 28—Novel foods” that regulates GMF. But novel food covers GMF and non-GMF considering the food characteristics not only the process of food production. Food characteristics refer to food composition, structure or nutritional quality; metabolism in the body; and, safety (microbiological, chemical and of use). Division 28 sets the meaning of novel food that comprises: (a) substance with no history of safe use as a food; (b) food obtained (manufactured, prepared, preserved, or packaged) through a process that has not been previously applied to food production and causes a major change; (c) food derived from genetically modified plant, animal, or microorganism.

Health Canada and the Canadian Food Inspection Agency (CFIA) shares responsibility for the regulation of products derived from biotechnology, including GMF labeling policies. The Health Canada develops policy and sets standards related to the health and safety aspects of labeling and the CFIA enforces the regulations and applies these specific policies and the general food labeling policies. Additionally, the CFIA assures the protection of the consumers from misrepresentation and fraud related to food labeling, packaging, and advertising. The Health Canada considers that special labeling is required to ensure the safe use of the food, such as major compositional or nutritional changes in the food. In this situation, the Health Canada will determine what type of information consumer needs to receive for health and safety reasons (Government of Canada, 2017).

In Canada, public opinion has been analyzed through surveys conducted in the last years by diverse organizations and the results confirmed that citizens support mandatory labeling of GMF. The most recent studies found that, in 2012, 91% of Canadians wanted mandatory labeling (Leger Marketing, 2012). In 2015, the number was 88% of Canadians (Ipsos Reid, 2015) and reached 78% of Canadians pro mandatory labeling in 2016 (The Strategic Counsel, 2016). Considering the public opinion and the position of some representative Canadian organizations, parliamentarians discussed updating the Foods and Drugs Act through the Bill C-291(Government of Canada, 2016). This bill proposed a mandatory labeling of GMF but it did not pass in 2017.

Consequently, there is no mandatory label for GMF in Canada justified by the process of production, on the contrary, health and safety reasons assessed by the Health Canada will determine specific labeling whether the food is GM or not. However, it is permitted to use voluntary labeling that must follow standard rules applied to all types of food.

## Consumers interest in information and consequences for food security

The information contained in a label is valuable, necessary, and a consumer right. The exercise of this right enables the consumer to learn about important product traits (Messer et al., 2015). However, in name of the “right to information,” labeling requirements may extend beyond what is reasonable and necessary. The guidelines of international institutions are clear on the kind of information that should be included on the food labels. Recommendation of GMOs and GMF labeling in Brazil, in our opinion, overstates the suggestions of International Institutions involved in Food Safety. Brazil is currently a major player in the commodities market (Confederação Nacional da Indústria, 2013), but if the country adopts an unscientific stance to deal with GMOs, it may lose competitiveness. GMF labeling involves special processes in the production chain in order to ensure that the GMO-containing products be segregated from other foodstuffs from cultivation to packaging. Thus, the current regulation requires procedures that increase significantly the costs of production, storage, and transportation which will be passed on to the final consumer.

Countries should avoid regulations that might create domestic and international trade barriers. Moreover, mandatory labeling should not conflict with the World Trade Organization (WTO) agreements. The WTO parties have agreed to treat equally similarly classified product. Differential labeling for commercially approved products obtained from GM plants is inconsistent with that policy. While the consensus in the scientific and farming communities is that GMF is safe, public opinion remains divided (de Carvalho Borges et al., 2009; Małyska et al., 2014; Capalbo et al., 2015; Han et al., 2015; Lucht, 2015; McFadden and Lusk, 2015). In many countries, surveys have shown that the public favors mandatory labeling of GMF, regardless of the risks involved (Premanandh, 2011), even when they are aware that growing genetically modified plants is only allowed after an extensive risk analysis and a governmental (bio)safety approval.

Why do consumers desire mandatory labeling of GMF in spite of substantial and growing evidence documenting its safety? The impact of information depends on prior beliefs. Several factors may conspire against revising one's opinion based on new information, including information misinterpretation, knowledge/cognition, political affiliation, illusory correlations, selective information acquisition, and information processing problems. As a result, many people place greater weight on non-scientific information or misinterpret the scientific information, convincing themselves that new information simply confirms their prior belief (McFadden and Lusk, 2015). Although people express an interest in obtaining certain information, this fact does not guarantee them the right to make their mandatory exposure on the label (MacDonald and Whellams, 2007).

The issue of the large companies and the impact of their patents on food security has also raised the discussion on mandatory labeling because consumers may associate big multinationals with GM (Blancke et al., 2015). Consumers may also be concerned about the potential environmental impact of GM crops because they intrinsically associate with pesticide use. While those are important topics, they should be addressed in their own rights, rather than using GMF labeling as an indirect proxy for what consumers are really concerned about. Studies have shown that growing GM crops may result in increased (Almeida et al., 2017) or reduced use of pesticides (Taheripour et al., 2016). Therefore, not all GM plants can be associated with this outcome. Accordingly, attempts to extend the concept of the risk assessment, especially in the European Union (EU), to include property aspects as well as general socio-economic factors (Smyth et al., 2015) inappropriately intertwines disparate issues with an attempt loss of precision in the debate.

The public outcry goes beyond scientific knowledge and cannot be appeased with more scientific information but only with confidence in the institutions (Małyska et al., 2014). Responding to the public clamor by legislating unreasonable rules regarding differential labeling creates an additional obstacle for the acceptance of GMF (Kaneko and Chern, 2005). This solution ignores risk analysis and reinforces a culture of insecurity and distrust in public institutions (McHughen and Wager, 2010). A more reasonable measure would be to avoid mandatory GMF labeling and take measures to restore confidence in government institutions, with appropriate communication strategies (Prati et al., 2012; Lang, 2013; Capalbo et al., 2015). In Australia, GMF labeling has been mandatory for a long time and the US has recently adopted although it is not in force yet. However, Canada is an example where government considered that GMF labeling is unjustified even though the public is claimoring for it.

If GMF producers themselves choose to accede to consumers' claims about GMF information, they have governance tools available, such as voluntary labeling and certification schemes. An interesting example is the case of the Roundtable for Sustainable Palm Oil (RSPO) certification, which has the purpose of achieving both ecological and food security goals in the palm oil production chain (Oosterveer et al., 2014).

The prospect of burdensome and prejudicial regulatory requirements has impaired the development of biotechnology products in universities and small businesses (Servick, 2015). In fact, the requirement for GMF mandatory labeling imposes additional obstacles to the technological development of genetic advances (Premanandh, 2011) despite the fact that this technology may increase the productivity in developing countries (Anderson, 2010), help subsistence and family agriculture. It also has the potential of avoiding diseases in vegetables and fruits and improves the nutritional value of regional foods such as rice and cassava (González et al., 2009).

Brazil, like other developing countries, has significant research capability in its public institutions. The development of GM beans carried out by the Brazilian Agricultural Research Corporation (EMBRAPA), a public research institution, provides an example of such expertise. Embrapa 5.1 is a common bean genetically manipulated to make it resistant to golden mosaic, a viral disease that significantly reduces productivity (Aragão et al., 2013; EMBRAPA, 2014). This project shows that GM will benefit small farmers because in Brazil, 80% of the bean production is grown in areas of < 100 hectares. This is a culture of extreme social relevance especially because beans are the main source of vegetable protein and a considerable source of iron in the country (República Federativa do Brasil, 2010).

## Conclusions

The world relies on international food trade to improve food security. In order to achieve this goal, countries need to strive to harmonize their regulations as closely as possible with CAC suggestions. GFAR provides an appropriate forum for discussions about GMOs and GMF. Using that forum to obtain global consensus about GMF labeling and develop proposals for updating countries' regulations would avoid the current unscientific approach and would help to remove a major impediment to the technological development.

People's attitude toward GMF labeling has been informed by non-scientific arguments and misinterpretation of scientific data. The resulting fear may be exploited to sustain companies' profits based on unclear and dishonest labels such as “GMO-free,” despite the fact that current scientific evidence finds no difference in food safety risk.

This conclusion does not infringe the right to information as it is based on the international recommendation for food labeling. These documents provide that if the risks are considered negligible by government technical agencies and the product is considered safe to be marketed and consumed, information about its GM nature is superfluous and should not be mandatory. In our opinion, the labeling policies currently practiced in Brazil are misleading. Use of the symbol containing the letter “T” in a yellow triangle may easily be interpreted as a sign of danger, although the product has been rigorously tested before being approved for commercial releasing. On the other hand, despite the lack of scientific evidence about the harm of the GMF and derivatives, if a political decision establishes the adoption of a GMO symbol, it should not imply danger. In order to illustrate, we have discussed about the characteristics of Radura.

The socioeconomic implications of the GMF issue have special importance in Brazil due to the pivotal role of the R&D in public institutions for the Brazilian innovation system. Since the 1980s, the great majority of agricultural research has been conducted in public institutions and has supported Brazil's leading position in world agriculture. The public investments in R&D have had a better return than have private investments in terms of patents and innovation during the last decades (De Negri et al., 2015). The interaction between public universities and the industrial sector is still small in Brazil (De Negri et al., 2015) and faces several challenges (Rapini et al., 2015). In this scenario, mandatoryGMF labeling ends up being a discriminatory action by the regulatory authorities because it indirectly raises doubts about risk assessment validity.

FAO exhorts governments to provide a rational, scientific basis for the regulation of biosafety and to strengthen their rural extension institutions (FAO, 2010a). While new regulations are often needed in the case of scientific breakthroughs for sustainable agriculture, these products are submitted to a rigorous risk analysis before being marketed. The first technology that had enabled genetic modifications, recombinant DNA technology, was developed in 1973 (Russo, 2003) and has just completed its 45th anniversary. Currently, we face a new generation of biotechnology products derived from new tools (e.g., gene editing systems). Therefore, the evolution of these technologies meets the backwards movement of genetically modified labeling policies. An international consensus has not been reached yet and Brazilian Congress seems to provide an eternal discussion about the GMF labeling, keeping it mandatory.

Finally, we consider that only a scientifically informed decision-making process will yield appropriate regulations crafted to improve food security, safety, and transparency.

## Author contributions

BB, OA, AF, JB, and PF have significantly contributed for elaboration, discussion, and revision of this work.

### Conflict of interest statement

The authors declare that the research was conducted in the absence of any commercial or financial relationships that could be construed as a potential conflict of interest.
